# Influence of Very Short-Term Sexual Abstinence on Basic Semen Parameters and Sperm Chromatin Status: A Preliminary Study

**DOI:** 10.3390/ijms27115046

**Published:** 2026-06-03

**Authors:** Kamil Gill, Mariusz Łukaszuk, Tomasz Machałowski, Małgorzata Piasecka

**Affiliations:** 1 Department of Histology and Developmental Biology, Faculty of Health Sciences, Pomeranian Medical University, 71-210 Szczecin, Poland; 2 Novique—Aesthetic and Anti-Aging Medicine Clinic, 80-255 Gdańsk, Poland; 3 Department of Perinatology, Obstetrics and Gynaecology, Faculty of Medicine, Pomeranian Medical University, 72-010 Police, Poland; tomasz.machalowski@pum.edu.pl

**Keywords:** spermatogenesis, sexual abstinence, sperm maturation, sperm DNA

## Abstract

Infertility is a rising global medical problem that affects 15–20% of reproductive-age couples. Importantly, a male factor contributes to this issue in 30–50% of cases. Abnormalities in semen parameters, particularly sperm genome integrity, are major causes of male infertility. Recent reports indicate that the duration of sexual abstinence is a significant and easily modifiable factor that may influence semen quality. Therefore, this study was designed to evaluate the effects of very short-term sexual abstinence on standard semen parameters and sperm chromatin status. This study was conducted on semen samples obtained from adult men (n = 95) who were partners in couples with reproductive failure. Each participant provided two semen samples on the same day: the first after 2–7 days of sexual abstinence and the second after one hour of sexual abstinence. Semen analysis was performed according to the World Health Organization (2021) guidelines, and the sperm chromatin status was determined using three complementary assays: sperm chromatin dispersion test (SCDt), and aniline blue (ABt) and toluidine blue (TBt) stains. One-hour sexual abstinence significantly reduced the following parameters: ejaculate volume (median: 3.50 mL vs. 1.50 mL), total sperm count (median: 78.30 × 10^6^ sperm vs. 29.00 × 10^6^ sperm cells), number of sperm cells with DNA fragmentation (SDF index; SCDt; median: 19.00% vs. 16.50%), prevalence of subjects with an SDF index > 20% (44.18% vs. 24.46%) and odds ratio for an SDF index > 20% (OR = 0.4568). No significant differences were found in the results of the ABt or TBt. Our data indicate that a very short period of sexual abstinence negatively affects ejaculate volume and total sperm count but positively influences sperm genome integrity. Importantly, the sperm chromatin dispersion test can be considered a useful tool for verifying sperm DNA quality.

## 1. Introduction

Approximately 15–20% of couples of reproductive age suffer from infertility (over 12-month period of unsuccessful attempts), and it is estimated that male factors are recognized as a cause in 30–50% of infertile couples [1]. One of the most frequently diagnosed cause of decreased male fertility potential is idiopathic infertility (approximately 30% of cases), characterized by abnormalities in standard sperm characteristics of unknown aetiology, which significantly complicates therapeutic management [2]. Some reports indicate that the duration of sexual abstinence can be an effective and easily modifiable factor improving sperm parameters. Moreover, adjusting the abstinence period appears to be a safe medical approach, as it does not carry the risks associated with pharmacological treatments such as antioxidant therapy, which may increase the risk of reductive stress in the absence of oxidative stress—a phenomenon known as the “antioxidant paradox” [3,4,5,6,7,8].

Modification of the duration of sexual abstinence influences the time of sperm transit through the epididymis. It is generally estimated that epididymal transit in humans takes approximately 10–15 days [9]. However, some authors suggest that the duration of epididymal transit may vary considerably between individuals. In some cases, this transit appears to be relatively rapid, lasting only a few days (1–2 days), whereas in others it may take 2–6 days or even 10–15 days. This variability contrasts with the more consistent 10–12-day transit reported for laboratory and domestic animals [10,11,12,13,14]. During this passage, nearly 90% of the luminal fluid is reabsorbed, leading to a marked increase in the sperm concentration. Notably, the epididymal epithelium is involved in the creation of an optimal environment for sperm maturation [9,10,15,16,17]. Epididymal maturation is associated with structural, functional, and molecular changes in sperm cells. For example, when spermatozoa lose cytoplasmic droplets, the acrosome becomes narrower, resulting in the formation of disulfide bridges in proteins, including protamines, which is related to the epididymal compaction of chromatin [9,18,19]. Finally, the sperm cells acquire the ability to undergo progressive motility. Furthermore, antioxidant enzymes, such as superoxide dismutase, are secreted into the epididymal lumen to counteract oxidative stress and protect spermatozoa against reactive oxygen species (ROS). This is essential because of the high metabolic activity and generation of ROS by mitochondria in epididymal epithelial cells (electron leakage at the respiratory chain level), as well as by immature sperm cells (especially those with residual cytoplasm rich in NADPH oxidase (NOX5)) and by leukocytes (through respiratory bursts) [9,10,17].

According to World Health Organization (WHO) guidelines [20], for standardization of the semen analysis, the recommended period of sexual abstinence before routine semen analysis is 2–7 days. However, the European Society of Human Reproduction and Embryology (ESHRE) recommends a shorter abstinence period of 3–4 days [21]. Some studies have shown that prolonged sexual abstinence increases sperm exposure to ROS and promotes apoptosis, leading to decreased sperm function (motility and vitality) and impaired genome integrity [5,6,7,8,9,22]. Only a few publications [4,5,23,24,25] have described the pros and cons of very short intervals between ejaculations (1–4 h) in dependent groups of men (the same subjects). Therefore, the aim of this study was to verify the influence of very short-term sexual abstinence on standard semen parameters as well as on sperm chromatin integrity and maturity.

## 2. Results

### 2.1. Basic Semen Analysis

**Figure 1:**
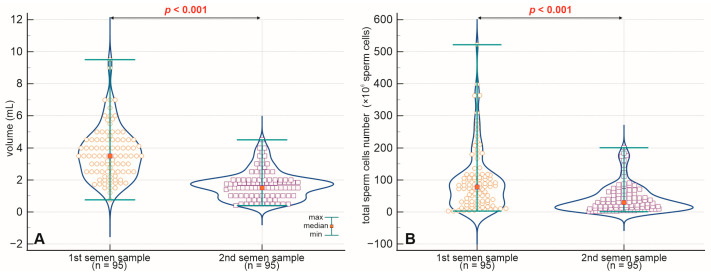
Comparison of the semen volume (**A**) and total sperm cell number (**B**) between the 1st semen sample (after 2–7 days of sexual abstinence) and the 2nd semen sample (after 1 h after sexual abstinence). The dependent groups of men were compared using the Wilcoxon test (median test); n, number of participants. A *p* value < 0.05 was considered to indicate statistical significance.

**Figure 2:**
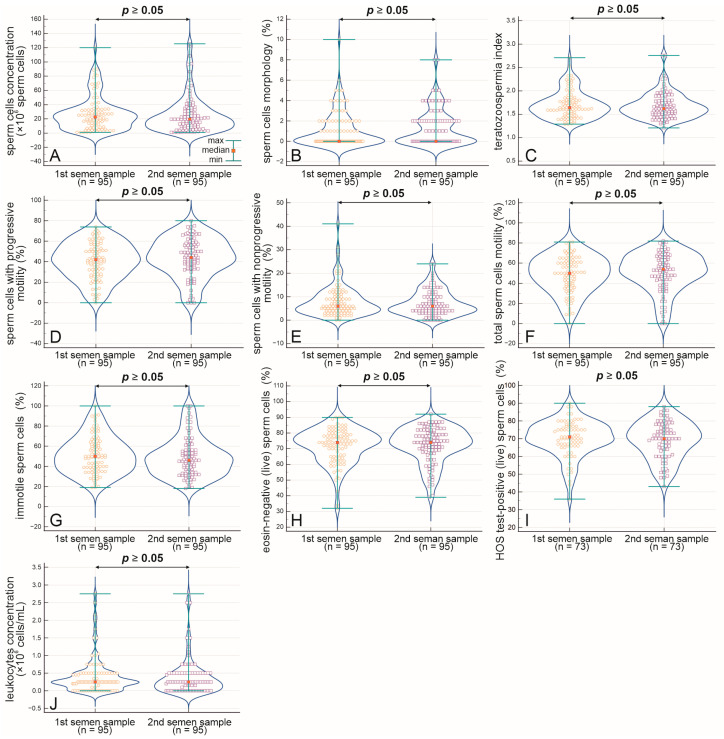
Comparisons of the sperm cell concentration (**A**), morphology (**B**), teratozoospermia index (**C**), sperm cell progressive motility (**D**), nonprogressive motility (**E**), total motility (progressive + nonprogressive motility) (**F**), immotile sperm cells (**G**), eosin-negative (live) sperm cells (**H**), HOS test-positive (live) sperm cells (**I**) and leukocyte concentration (**J**) between the 1st semen sample (after 2–7 days of sexual abstinence) and the 2nd semen sample (after 1 h of sexual abstinence). The dependent groups of men were compared using the Wilcoxon test (median test); HOS test, hypo-osmotic swelling test; n, number of participants. A *p* value < 0.05 was considered to indicate statistical significance.

Comparison of standard semen parameters between the first (after 2–7 days of sexual abstinence) and the second (after 1 h of sexual abstinence) semen samples revealed a significant influence of very short-term sexual abstinence on the semen volume (median: 3.50 mL vs. 1.50 mL; Hodges–Lehmann estimator −1.88 mL) (Figure 1A; Supplementary Table S1) and total sperm cell number (median: 78.30 × 10^6^ sperm cells vs. 29.00 × 10^6^ sperm cells; Hodges–Lehmann estimator: −43.79 × 10^6^ sperm cells) (Figure 1B; Supplementary Table S2). However, there were no significant differences in terms of the sperm cell concentration (median: 22.41 × 10^6^ sperm cells/mL vs. 19.50 × 10^6^ sperm cells/mL; Hodges–Lehmann estimator: −1.33 × 10^6^ sperm cells/mL) (Figure 2A; Supplementary Table S3), sperm cell morphology (median: 0.00% vs. 0.00%; Hodges–Lehmann estimator: 0.00%) (Figure 2B; Supplementary Table S4), teratozoospermia index (TZI) (median: 1.64 vs. 1.62; Hodges–Lehmann estimator: −0.025) (Figure 2C; Supplementary Table S5), sperm cell progressive motility (median: 42.00% vs. 44.00%; Hodges–Lehmann estimator: 2.50%) (Figure 2D; Supplementary Table S6), sperm cell nonprogressive motility (median: 6.00% vs. 6.00%; Hodges–Lehmann estimator: −0.50%) (Figure 2E; Supplementary Table S7), or total sperm cell motility (median: 50.00% vs. 54.00%; Hodges–Lehmann estimator: 2.00%) (Figure 2F; Supplementary Table S8), immotile sperm (median: 50.00% vs. 46.00%; Hodges–Lehmann estimator: −2.00%) (Figure 2G; Supplementary Table S9), eosin-negative (live) sperm cells (median: 74.00% vs. 74.00%; Hodges–Lehmann estimator: 0.00%) (Figure 2H; Supplementary Table S10), hypo-osmotic swelling test (HOS test)-positive (live) sperm cells (median: 71.00% vs. 70.00%; Hodges–Lehmann estimator: −1.00%) (Figure 2I; Supplementary Table S11) or leukocyte concentration (median: 0.25 × 10^6^ cells/mL vs. 0.25 × 10^6^ cells/mL; Hodges–Lehmann estimator: 0.00 × 10^6^ cells/mL) (Figure 2J; Supplementary Table S12).

### 2.2. Sperm Chromatin Quality

**Figure 3:**
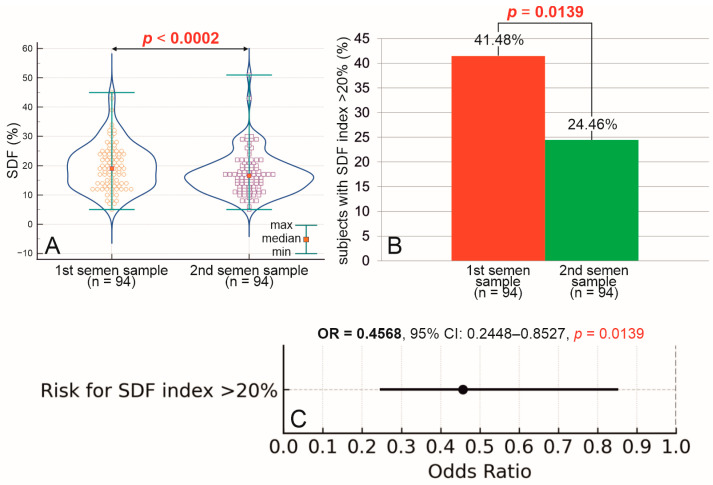
Comparison of the sperm DNA fragmentation index (SDF index) (**A**) as well as the prevalence of subjects with an SDF index > 20% (**B**) between the 1st semen sample (after 2–7 days of sexual abstinence) and the 2nd semen sample (after 1 h after sexual abstinence) and odds ratio (OR) for an SDF index > 20% after 1 h of sexual abstinence with respect to the first semen sample (**C**). The SDF values were compared using the Wilcoxon test (median test), while the chi2 test was used to determine the significance of differences in the prevalence of men with an SDF index > 20%; 95% CI, 95% confidence interval; n, number of participants. A *p* value < 0.05 was considered to indicate statistical significance.

**Figure 4:**
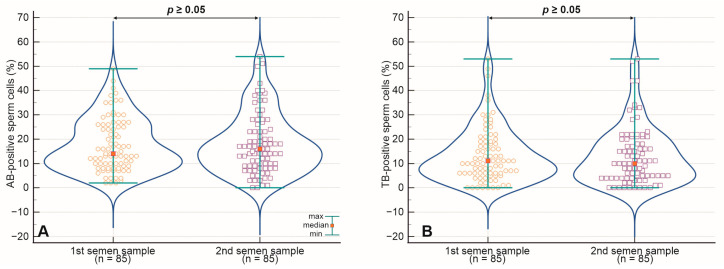
Comparison of the AB index (**A**) and TB index (**B**) between the 1st semen sample (after 2–7 days of sexual abstinence) and the 2nd semen sample (after 1 h after sexual abstinence). The dependent groups of men were compared using the Wilcoxon test (median test); AB, aniline blue, n, number of participants, TB, toluidine blue. A *p* value < 0.05 was considered to indicate statistical significance.

To comprehensively assess the effects of very short-term sexual abstinence on sperm chromatin quality/maturity sperm chromatin dispersion (SCDt), aniline blue (ABt) and toluidine bleu tests (TBt) were performed. Comparison of the obtained data revealed a significantly lower number of sperm cells with DNA fragmentation (SDF index) in the second semen sample (median: 19.00% vs. 16.50%; Hodges–Lehmann estimator: −2.00%) (Figure 3A; Supplementary Table S13), a significant decrease in the percentage of subjects with an SDF index > 20% (44.18% vs. 24.46%, difference: −19.72%) (Figure 3B) and an over 2-fold lower odds ratio (OR = 0.4568) for an SDF index > 20% (Figure 3C). There was no significant influence of the duration of sexual abstinence on the percentage of sperm cells exhibiting abnormal histone retention (AB index) (median: 14.00% vs. 16.00%; Hodges–Lehmann estimator: 0.50%) (Figure 4A; Supplementary Table S14) or the percentage of sperm cells with abnormal chromatin condensation (TB index) (median: 11.00% vs. 10.00%; Hodges–Lehmann estimator: −0.50%) (Figure 4B; Supplementary Table S15).

## 3. Discussion

Numerous studies have indicated that a prolonged period without ejaculation negatively affects semen parameters such as motility, vitality, and, crucially, sperm nuclear DNA integrity. Therefore, some authors recommend a period of sexual abstinence closer to 2 days rather than 7 days, especially in the context of the efficiency of assisted reproductive technology (ART) procedures [6,7,8,22,26]. Notably, most recent publications on the effect of sexual abstinence on semen parameters consider abstinence duration measured in days in independent groups of patients (different groups of subjects) [6,7,8,27,28,29]. Therefore, in this publication, we decided to focus on the influence of very short-term sexual abstinence (only one hour) on selected semen parameters, including a comprehensive assessment of sperm chromatin status.

### 3.1. Very Short Duration of Sexual Abstinence and Basic Semen Parameters

As expected, our results indicated that a very short duration of sexual abstinence (one hour) contributes to a significant decrease in ejaculate volume, which is consistent with the results of other authors [5,6,7,8]. We can assume that the decrease in semen volume resulted in a significantly reduced total number of spermatozoa. These findings have also been confirmed by other researchers [5,6,7,8,28]. However, no significant differences in the sperm concentration following one hour of sexual abstinence were observed in our study. We noted only a decreasing trend in this parameter (median: 22.41 × 10^6^ sperm cells/mL vs. 19.50 × 10^6^ sperm cells/mL). Other authors have shown that a shorter duration of sexual abstinence causes a decrease in the sperm concentration [6,7,8,25,27,29]. This decline can result from insufficient absorption of epididymal fluid during very short-term sexual abstinence [9,15]. Moreover, in our study, no significant changes in sperm morphology or the TZI were observed. Similarly, other researchers have demonstrated that modifying the duration of sexual abstinence is not a significant causative factor influencing sperm morphological features. Since sperm morphology primarily depends on testicular spermatogenesis, it is not significantly influenced by incidental changes in the duration of sexual abstinence [5,6,7,8]. However, Russo et al. [29] reported that in normozoospermic patients with 7 days of sexual abstinence, the number of sperm cells with normal morphology was significantly higher than that after 1 day, but it should be noted that this research was conducted on independent groups of men. It is also worth noting that the effect of a very short period of sexual abstinence (1 h) may depend on the semen phenotype. This was demonstrated by Manna et al. [30], who showed that, in group of normozoospermic men, very short-term sexual abstinence significantly increased the percentage of spermatozoa with normal morphology. In contrast, in group of oligoasthenozoospermic men, additionally a significant increase in the percentage of sperm cells with progressive motility was observed.

Unfortunately, despite our initial assumptions, a significant improvement in sperm motility after one hour of sexual abstinence was not observed. However, slight improvement in sperm progressive motility was noted (from 42% to 44%). Nevertheless, other authors [5,28] who analysed samples from the same patients on the same day also reported no significant influence of very short periods of sexual abstinence on the progressive motility of sperm. These data are interesting because the deterioration of sperm motility and vitality is most likely associated with the extensive exposure of sperm to reactive oxygen species and with increased apoptosis of male reproductive cells during prolonged sperm epididymal transit [6,7,8,25,29,31,32]. Moreover, regardless of time without ejaculation, oxidative stress (OS) in semen is associated with impaired semen quality, including reduced sperm motility, and may contribute to male infertility [33,34,35,36]. Hence, modifying the duration of sexual abstinence could provide an opportunity to significantly improve sperm motility and vitality by reducing the risk of oxidative stress and apoptosis in the epididymis [22,24,37]. On the other hand, as mentioned earlier, testicular spermatozoa are unable to undergo progressive motility, and sufficient time for epididymal maturation is necessary for sperm to acquire this ability [9,15,16].

The last of the assessed basic semen parameters was the leukocyte concentration. In our study, very short-term sexual abstinence did not significantly affect the concentration of peroxidase-positive cells. Notably, the men in our study had relatively low leukocyte concentrations (median 0.25 × 10^6^ cells/mL). Frequent ejaculation is recommended in cases of prostatitis to remove, among other factors, leukocytes and proinflammatory cytokines from the male reproductive system [38,39]. We can only speculate that in men with leukocytospermia, a shorter period of sexual abstinence might have a greater effect, resulting in a significant decrease in the number of inflammatory cells in the semen.

### 3.2. Very Short Duration of Sexual Abstinence and Sperm Chromatin Status

Notably, we clearly showed a dependency between a very short duration of sexual abstinence and sperm genomic integrity because we noted not only a decline in the median SDF index from 19.00% in the first sample to 16.50% in the second sample but also an almost two-fold reduction in the percentage of samples with an SDF index > 20% (from 44.18% to 24.46%). Consequently, OR analysis revealed an over two-fold decrease in the risk for an SDF index > 20% (OR: 0.4568) after very short-term sexual abstinence. It is worth noting that this threshold (20% SDF index) is currently recognized as the optimal value for predicting male fertility (to distinguish fertile men from infertile men) [40,41,42,43,44].

Similar results have been reported by other authors, who reported duration-dependent improvements in human sperm genome integrity [6,7,8,24,27,29,30]. We speculate that the sexual abstinence-dependent decrease in sperm DNA fragmentation may result from the shortening of the exposure time of the sperm to epididymal ROS. The epididymis is known to be a source of ROS generation [45].

On the other hand, our comprehensive analysis of sperm chromatin status did not reveal any significant effect of very short-term abstinence on the results of the ABt and TBt. The former test reveals the presence of residual nuclear histones in sperm chromatin, whereas the latter identifies chromatin structure abnormalities associated with accessible phosphate groups in immature sperm chromatin [46]. These results are not surprising because fundamental chromatin remodelling first occurs in the testes during spermiogenesis—the differentiation of round (early) spermatids to elongated (late) spermatids. This process involves the replacement of histones with sperm-specific chromatin proteins—protamines. This is required to compact genetic material to protect the sperm genome during the passage of male gametes through the male and female reproductive tracks [47,48]. Furthermore, final chromatin condensation (stabilization of chromatin structure—the formation of disulfide bridges in protamines) continues during epididymal sperm maturation [9,15,16]. Therefore, it is not surprising that we did not observe changes in the AB and TB indices. Additionally, other researchers [7] reported similar results with a sperm chromatin structure assay (SCSA). They exhibited associations between sperm DNA integrity and the duration of sexual abstinence, but no differences in chromatin structure/maturity were detected, as verified by a high DNA stainability (HDS) assay.

## 4. Materials and Methods

### 4.1. Participants

This study was carried out on semen samples obtained from adult participants (n = 95, median age: 33.00 years; range: 23.00–48.00 years) who were admitted to the Andrology Laboratory in the Department of Histology and Developmental Biology (Faculty of Health Sciences, Pomeranian Medical University in Szczecin, Poland). All the participants self-reported that they were partners in couples with reproductive failure (partners in couples who had not achieved pregnancy during at least 12 months of regular intercourse without contraception). Patients with cryptozoospermia or azoospermia were excluded from the study. The Ethics Committee of Pomeranian Medical University in Szczecin (Poland) approved the study protocol (ethical authorization number: KB-0012/43/2021). The subjects signed an informed consent form agreeing to take part in the research grant, in accordance with the Declaration of Helsinki.

### 4.2. Semen Sample Collection

The first semen sample of each participant was collected by masturbation after 2–7 days of sexual abstinence (median: 4 days, rang: 2–7 days, Q1: 3 days, Q3: 5 days, IQR: 2 days; 2 men reported 2 days of sexual abstinence, 28 men reported 3 days, 33 men reported 4 days, 18 men reported 5 days, 7 men reported 6 days, and 7 men reported 7 days) into a wide-mouth sterile plastic graded container (according to the 5th and 6th editions of the WHO guidelines [20,49]). The second semen sample was collected on the same day after 1 h of sexual abstinence. Both samples were collected in the laboratory and stored at 37 °C until the analyses were performed.

### 4.3. Assessment of Basic Semen Parameters

In accordance with the WHO guidelines [20], semen analysis was performed using fully liquefied samples (37 °C, 30 min). The following macroscopic parameters were evaluated: semen volume, liquefaction time, pH, and viscosity. In turn, the microscopic examination included the following: sperm concentration, motility, morphology, and viability and the leukocyte concentration. The light/phase-contrast microscope (DM500—Leica, Heerbrugg, Switzerland) was applied to assess the sperm concentration (×10^6^ sperm cells/mL; an improved Neubauer counting chamber—Heinz Hernez Medizinalbedarf GmbH, Hamburg, Germany) in diluted semen and sperm motility in native semen (both at 400× magnification). The progressive motility (sperm moving in a mostly straight line or in wide circles), nonprogressive motility (any other movement), and immotile spermatozoa were distinguished. Total sperm motility was calculated as the sum of spermatozoa with progressive and nonprogressive motility. In turn, sperm viability was evaluated using two tests: the eosin test and the HOS test. Eosin-negative sperm cells and hypo-osmotically reactive sperm cells were considered viable. Both tests were performed under bright-field microscopy at 1000× magnification and an oil immersion lens. Sperm morphology (Papanicolaou staining) was calculated according to Strict Criteria recommended by the WHO [20] under a bright-field microscope with 1000× magnification and an oil immersion lens. Additionally, based on number of spermatozoa with at least one defect in the head, midpiece, and tail, as well as the presence of immature spermatozoa with residual cytoplasm, the TZI was calculated. The TZI indicates the average number of morphological abnormalities per spermatozoon. The leukocyte concentration (peroxidase-positive cells) was determined by means of the Endtz test (LeucoScreen Kit, FertiPro N.V., Beernem, Belgium). Leukocytes were quantified (×10^6^ cells/mL) using an improved Neubauer counting chamber at 400× magnification under phase contrast illumination.

On the basis of the 5th centile (the lower reference limit) of standard semen parameters derived from fertile men whose partners achieved pregnancy within 12 months of attempting to conceive naturally [20,50,51], the following groups of men were distinguished:

- Eleven men had a total sperm count, progressive motility, and morphology ≥ the 5th centile (≥39.00 × 10^6^ sperm cells per ejaculate, ≥30% progressively motile sperm cells, and ≥4% sperm cells with normal morphology, respectively). This group was classified as normozoospermic (N) (median of sexual abstinence: 4 days, rang: 3–5 days, Q1: 3 days, Q3: 5 days, IQR: 2 days).
- Forty-five men had isolated sperm morphology < the 5th centile. This group was classified as teratozoospermic (T) median of sexual abstinence: 4 days, rang: 2–7 days, Q1: 3 days, Q3: 5 days, IQR: 2 days).
- Nine men had both progressive motility and morphology of sperm < the 5th centile. This group was classified as asthenoteratozoospermic (AT) median of sexual abstinence: 4 days, rang: 3–7 days, Q1: 4 days, Q3: 4 days, IQR: 0 days).
- Twelve men had both sperm count and morphology < the 5th centile. This group was classified as oligoasthenozoospermic (OT) median of sexual abstinence: 4 days, rang: 3–6 days, Q1: 3 days, Q3: 4.5 days, IQR: 1.5 days).
- Eighteen men had sperm count, progressive motility, and morphology all < the 5th centile. This group was classified as oligoasthenoteratozoospermic (OAT) median of sexual abstinence: 4 days, rang: 2–7 days, Q1: 3 days, Q3: 5 days, IQR: 2 days).

### 4.4. Assessment of Sperm DNA Fragmentation (SDF) with the Sperm Chromatin Dispersion Test (SCDt)

The chromatin dispersion test (SCDt) is used to assess genome integrity by evaluating the susceptibility of the sperm nucleus to dispersed DNA loop formation. This test was performed using a Halo-sperm G2^®^ kit (Halotech, Madrid, Spain). Sample preparation followed the manufacturer’s instructions provided with the kit. Briefly, the recommended sperm concentration for the assay should not exceed 20 × 10^6^ cells/mL. If necessary, the semen samples were diluted in phosphate-buffered saline (PBS). The semen sample was gently mixed with melted agarose (in proportion 1:2, 37 °C). Subsequently, 8 µL of the suspension was dropped onto a supercoated slide and covered with a coverslip (4 °C, 5–10 min). After removing of the coverslip, the semen sample was treated with acid denaturation solution (DA) (room temperature, 7 min). In the next step, the DA was discarded and lysis solution (LS) was used (room temperature, 20 min) which following incubation was removed. The slides were then rinsed off with distilled water (room temperature, 5 min), dehydrated sequentially in 70% and 100% ethanol (room temperature, 2 min), and stained with eosin staining solution A and thiazine staining solution B (room temperature, 7 min).

Sperm DNA fragmentation (SDF) was evaluated manually using a brightfield microscope at 400× magnification (DM500 light microscope; Leica, Heerbrugg, Switzerland). An intact sperm genome was indicated by the presence of a visible halo of DNA loops surrounding the sperm head. In SCDt, SDF-negative spermatozoa were defined as those with halos greater than one-third of the core (related to the sperm head) diameter (medium halos) or halos larger than the core diameter (large halos). Spermatozoa exhibiting small halos, no halos, and irregular or weakly stained cores (degraded chromatin) were classified as SDF positive. The results are expressed as the proportion of SDF-positive cells relative to the total number of assessed spermatozoa and multiplied by 100. Therefore, the SDF index denotes the percentage of sperm with damaged/fragmented DNA. In accordance with the manufacturer’s protocol, the cut-off value for the SDF index is 20% for intrauterine insemination and 25% for in vitro fertilization procedures. According to most researchers, the threshold of 20% is considered the most useful for distinguishing between fertile and infertile men [40,41,42,43,44].

### 4.5. Assessment of Persistent Histones in the Sperm Chromatin: Aniline Blue Test (ABt)

Aniline blue is an acidic dye that selectively binds to lysine-rich histones in the sperm nucleus. This staining method enables the identification of spermatozoa with abnormally retained histones, which may adversely affect chromatin condensation within the sperm nucleus [46,52]. As such, sperm nuclei with loose chromatin packaging take up the stain and appear dark blue when viewed under a bright field microscope. Briefly, native semen samples (300–500 μL) were centrifuged at 600× *g* for 10 min. The supernatant was discarded, and the pellet was washed twice with 1 mL of PBS (centrifugation at 600× *g* for 10 min each). Air-dried smears were then fixed in 3% (*v*/*v*) buffered glutaraldehyde in PBS (pH 7.2) for 30 min. Following fixation, the smears were stained with 5% (*w*/*v*) aqueous aniline blue solution (Sigma-Aldrich Co., St. Louis, MO, USA) prepared in 4% acetic acid (pH 3.5) for 5 min. Then, the slides were rinsed with distilled water. Spermatozoa were evaluated under a bright-field microscope at 400× magnification using an oil immersion lens. The percentage of sperm cells exhibiting abnormal histone retention (aniline blue-positive spermatozoa) was calculated as the ratio of dark blue-stained sperm heads (partial or complete staining, indicating lysine-rich histones) to the total number of sperm heads analysed (both dark blue-stained and pale blue-stained, the latter representing mature chromatin), multiplied by 100 (AB index) [46].

### 4.6. Assessment of Abnormal Sperm Chromatin Condensation: Toluidine Blue Test (TBt)

Toluidine blue is a metachromatic dye that binds to DNA at sites with accessible phosphate groups. This staining method allows the identification of spermatozoa with decondensed chromatin, which may indicate compromised chromatin integrity within the sperm nucleus [46,53]. Native semen samples (300–500 μL) were centrifuged at 600× *g* for 10 min. The supernatant was discarded, and the pellet was washed twice with 1 mL of phosphate-buffered saline (PBS; pH 7.4) by centrifugation at 600× *g* for 10 min. Air-dried smears were fixed in freshly prepared 96% (*v*/*v*) ethanol–acetone solution (1:1, *v*/*v*) at 4 °C for 30 min and subsequently hydrolysed in 0.1 mol/L hydrochloric acid (HCl) at 4 °C for 5 min. After hydrolysis, the slides were rinsed three times in distilled water for 2 min each and then stained with 0.05% (*w*/*v*) toluidine blue (TB; Sigma-Aldrich Co., St. Louis, MO, USA) prepared in 50% McIlvaine’s citrate–phosphate buffer (pH 3.5) for 11 min at room temperature. The slides were then rinsed again in distilled water. Spermatozoa were evaluated under a bright-field microscope at 400× magnification using an oil immersion lens. The percentage of sperm cells with abnormal chromatin condensation (TB-positive cells) was calculated as the ratio of the number of spermatozoa with dark blue or deep violet (purple) staining (partial or complete staining of the sperm head) to the total number of spermatozoa analysed (including both TB-positive and pale blue-stained sperm heads with mature chromatin), multiplied by 100 (TB index) [46].

### 4.7. Statistics

The normality of continuous variables was assessed using the Shapiro–Wilk test. As the assumption of a normal distribution was rejected, comparisons between paired observations (the results of semen analysis from the first sample vs. the results of semen analysis from the second sample) were conducted using the nonparametric Wilcoxon rank test. Descriptive statistics included medians, 95% confidence intervals (95% CI) for medians, minimum and maximum values, quartile (Q), interquartile ranges (IQR), differences in medians (estimated using the Hodges–Lehmann estimator [median differences]), and two-sided *p* values for statistical significance. On the basis of the cut-off value of the SDF index (20%), the prevalence of subjects with an SDF index > 20% was calculated and compared between the two dependent groups using the chi-square test. Additionally, odds ratios (ORs) with 95% confidence intervals were calculated to assess the likelihood of an SDF index > 20% in the second sample relative to the first sample. A *p* value < 0.05 was considered to indicate statistical significance for all tests. Statistical analyses were performed using MedCalc version 22.009 (MedCalc Software Ltd., Ostend, Belgium) and Statistica version 13.0 (StatSoft, Cracow, Poland).

## 5. Strengths and Limitations of the Study

In our opinion, this research project, which examined the effects of very short-term sexual abstinence on semen parameters and sperm chromatin status, provides interesting and novel data that may be useful in the diagnostic and therapeutic management of infertility, particularly in the context of the increasing prevalence of male reproductive disorders. The present study demonstrated that a simple, safe, and cost-effective modification, like shortening the duration of sexual abstinence to one hour, may improve the genomic integrity of human sperm cells. Additionally, the strengths of this study include the relatively large number of participants, which allowed reliable statistical analyses to be performed, the use of a dependent-group study design, and the complement of standard semen analysis with a comprehensive assessment of sperm chromatin status using ABt, TBt, and SCDt. However, we are aware of certain limitations of the study. These undoubtedly include the heterogeneity of the study group, reflected both by differences in the duration of sexual abstinence before collection of the first semen sample (IQR: 2 days) and by the inclusion of men with different semen phenotypes, including normozoospermia as well as abnormalities in sperm count, motility, and morphology.

## 6. Conclusions

Our data indicate that, on the one hand, a very short period of sexual abstinence negatively affects ejaculate volume and total sperm count, which is fully understandable. On the other hand, it has a positive influence on sperm DNA integrity (the SDF index). The obtained chromatin integrity results show that the sperm chromatin dispersion test can be considered a useful tool for verifying sperm DNA quality. This conclusion is in line with most recent scientific papers and current trends in the diagnosis of male infertility [40,41,42,54,55,56].

Moreover, our results have practical implications, particularly for in vitro fertilization treatments. In the case of natural conception and intrauterine insemination (IUI), a very short period of sexual abstinence seems unjustified because reproductive success is partially dependent on a large number of spermatozoa in the ejaculate [57]. In the case of in vitro fertilization, the integrity of the sperm genome plays a particularly important role. Reduced sperm chromatin integrity is known to have detrimental effects on fertilization, early embryo development, implantation, and even offspring health and can be a risk factor for miscarriage. Therefore, we believe that very short-term sexual abstinence, which reduces sperm DNA fragmentation, could significantly improve in vitro fertilization outcomes. Notably, it is an effective, cost-free, and rapid approach for the management of DNA fragmentation in male reproductive cells. It may be worth considering the inclusion of abstinence-duration modifications in therapeutic algorithms [22,26,41,42,56,58,59,60].

## Data Availability

The raw data supporting the conclusions of this article will be made available by the authors on request.

## Supplementary Materials

The following supporting information can be downloaded at: https://www.mdpi.com/article/10.3390/ijms27115046/s1.

## Abbreviations

The following abbreviations are used in this manuscript:

AB	aniline blue
ABt	aniline blue test
ART	assisted reproductive technology
AT	asthenoteratozoospermia
CI	confidence interval
DA	denaturation solution
DNA	deoxyribonucleic acid
ESHRE	European Society of Human Reproduction and Embryology
HCl	hydrochloric acid
HDS	high DNA stainability
HOS	hypo-osmotic swelling
ICSI	intracytoplasmic sperm injection
IUI	intrauterine insemination
IQR	interquartile range
LS	lysis solution
N	normozoospermia
NADPH	nicotinamide adenine dinucleotide phosphate
NOX5	NADPH oxidase 5
OAT	oligoasthenoteratozoospermia
OR	odds ratio
OS	oxidative stress
OT	oligoteratozoospermia
PBS	phosphate-buffered saline
Q1	first quartile
Q3	third quartile
ROS	reactive oxygen species
SCD	sperm chromatin dispersion
SCDt	sperm chromatin dispersion test
SCSA	sperm chromatin structure assay
SDF	sperm DNA fragmentation
T	teratozoospermia
TB	toluidine blue
TBt	toluidine blue test
TZI	teratozoospermia index
WHO	World Health Organization
