# Supplementary material for: Influence of Very Short-Term Sexual Abstinence on Basic Semen Parameters and Sperm Chromatin Status: A Preliminary Study
Source: Int J Mol Sci. 2026 Jun 3;27(11):5046. doi: 10.3390/ijms27115046 (PMC13256629; doi:10.3390/ijms27115046)
Supplement: Supplementary file 1 [file ijms-27-05046-s001.zip › ijms-4343192-supplementary.pdf]

Supplementary Tables:

Supplementary Table S1. Descriptive statistics and comparisons of the semen volume between the 1st semen sample (after 2–7 days of sexual abstinence) and the 2nd semen sample (after 1 hour of sexual abstinence).

Variable	Semen volume of the 1st semen sample	Semen volume of the 2nd semen sample
Sample size (n)	95	95
**Median (mL)**	**3.50**	**1.50**
95% CI for the median (mL)	3.00–4.00	1.50–1.80
Lowest value (mL)	0.75	0.40
Highest value (mL)	9.50	4.50
IQR (mL)	2.50–4.50	1.00–2.00
Hodges-Lehmann median difference (mL)	–1.88	
95%CI Hodges-Lehmann median difference (mL)	–2.20—1.63	
Two-tailed probability (*p*)	**< 0.001**	

Notes: The groups were compared using the Wilcoxon test (median test).
Abbreviations: 95% CI, 95% confidence interval; IQR, interquartile range; n, number of subjects; p, level of statistical significance of differences between the study groups.

Supplementary Table S2. Descriptive statistics and comparisons of the total number of sperm cells between the 1st semen sample (after 2–7 days of sexual abstinence) and the 2nd semen sample (after 1 hour of sexual abstinence).

Variable	Total number of sperm cells of the 1st semen sample	Total number of sperm cells of the 2nd semen sample
Sample size (n)	95	95
**Median (×10⁶)**	**78.30**	**29.00**
95% CI for the median ($\times10^6$)	57.58–87.90	19.35–36.29
Lowest value ($\times10^6$)	2.42	0.60
Highest value ($\times10^6$)	522.00	200.73
IQR ($\times10^6$)	30.15–115.94	10.00–56.72
Hodges-Lehmann median difference ($\times10^6$)	–43.79	
95%CI Hodges-Lehmann median difference ($\times10^6$)	–56.72—30.28	
Two-tailed probability (*p*)	**< 0.0001**	

Notes: The groups were compared using the Wilcoxon test (median test).
Abbreviations: 95% CI, 95% confidence interval; IQR, interquartile range; n, number of subjects; p, level of statistical significance of differences between the study groups.

Supplementary Table S3. Descriptive statistics and comparisons of the sperm concentration between the 1st semen sample (after 2–7 days of sexual abstinence) and the 2nd semen sample (after 1 hour of sexual abstinence).

Variable	Sperm concentration of the 1st semen sample		Sperm concentration of the 2nd semen sample
Sample size (n)	95		95
**Median (×10⁶/mL)**	22.41		19.50
95% CI for the median (×10⁶/mL)	18.33–26.44		14.78–22.15
Lowest value (×10⁶/mL)	1.05		0.95
Highest value (×10⁶/mL)	120.00		125.50
IQR (×10⁶/mL)	2.50–4.50		1.00–2.00
Hodges-Lehmann median difference (×10⁶/mL)		–1.33	
95%CI Hodges-Lehmann median difference (×10⁶/mL)		–4.65–1.28	
Two-tailed probability (*p*)		0.3448	

Notes: The groups were compared using the Wilcoxon test (median test).
Abbreviations: 95% CI, 95% confidence interval; IQR, interquartile range; n, number of subjects; p, level of statistical significance of differences between the study groups.

Supplementary Table S4. Descriptive statistics and comparisons of the sperm cell morphology between the 1st semen sample (after 2–7 days of sexual abstinence) and the 2nd semen sample (after 1 hour of sexual abstinence).

Variable	Sperm cell morphology of the 1st semen sample		Sperm cell morphology of the 2nd semen sample
Sample size (n)	95		95
**Median (%)**	0.00		0.00
95% CI for the median (%)	0.00–1.00		0.00–2.00
Lowest value (%	0.00		0.00
Highest value (%)	10.00		8.00
IQR (%)	0.00–2.00		0.00–2.00
Hodges-Lehmann median difference (%)		0.00	
95%CI Hodges-Lehmann median difference (%)		0.00–0.00	
Two-tailed probability (*p*)		0.2676	

Notes: The groups were compared using the Wilcoxon test (median test).
Abbreviations: 95% CI, 95% confidence interval; IQR, interquartile range; n, number of subjects; p, level of statistical significance of differences between the study groups.

Supplementary Table S5. Descriptive statistics and comparisons of the teratozoospermia index (TZI) between the 1st semen sample (after 2–7 days of sexual abstinence) and the 2nd semen sample (after 1 hour of sexual abstinence).

Variable	TZI of the 1st semen sample	TZI of the 2nd semen sample
Sample size (n)	95	95
**Median**	1.64	1.62
95% CI for the median	1.60–1.71	1.58–1.68
Lowest value	1.29	1,21
Highest value	2.71	2,76
IQR	1.48–1.83	1.50–1.80
Hodges-Lehmann median difference	–0.025	
95%CI Hodges-Lehmann median difference	–0.06–0.01	
Two-tailed probability	0.1582	

Notes: The groups were compared using the Wilcoxon test (median test).
Abbreviations: 95% CI, 95% confidence interval; IQR, interquartile range; n, number of subjects; p, level of statistical significance of differences between the study groups.

Supplementary Table S6. Descriptive statistics and comparisons of sperm cell progressive motility between the 1st semen sample (after 2–7 days of sexual abstinence) and the 2nd semen sample (after 1 hour of sexual abstinence).

Variable	Sperm cells progressive motility of the 1st semen sample	Sperm cells progressive motility of the 2nd semen sample
Sample size (n)	95	95
**Median (%)**	42.00	44.00
95% CI for the median (%)	37.47–46.00	40.00–50.00
Lowest value (%)	0.00	0.00
Highest value (%)	74.00	80.00
IQR (%)	28.00–54.50	33.25–58.75
Hodges-Lehmann median difference (%)	2.50	
95%CI Hodges-Lehmann median difference (%)	–1.50–6.00	
Two-tailed probability	0.1504	

Notes: The groups were compared using the Wilcoxon test (median test).
Abbreviations: 95% CI, 95% confidence interval; IQR, interquartile range; n, number of subjects; p, level of statistical significance of differences between the study groups.

Supplementary Table S7. Descriptive statistics and comparisons of the nonprogressive motility of sperm cells between the 1st semen sample (after 2–7 days of sexual abstinence) and the 2nd semen sample (after 1 hour of sexual abstinence).

**Variable**	**Sperm cells nonprogressive motility of the 1st semen sample**	**Sperm cells nonprogressive motility of the 2nd semen sample**
Sample size (n)	95	95
**Median (%)**	6.00	6.00
95% CI for the median (%)	5.00–8.00	5.00–7.00
Lowest value (%)	0.00	0.00
Highest value (%)	41.00	24.00
IQR (%)	4.00–11.00	4.00–10.00
Hodges-Lehmann median difference (%)	–0.50	
95%CI Hodges-Lehmann median difference (%)	–1.50–0.50	
Two-tailed probability	0.4594	

Notes: The groups were compared using the Wilcoxon test (median test).
Abbreviations: 95% CI, 95% confidence interval; IQR, interquartile range; n, number of subjects; p, level of statistical significance of differences between the study groups.

Supplementary Table S8. Descriptive statistics and comparisons of total sperm cell motility between the 1st semen sample (after 2–7 days of sexual abstinence) and the 2nd semen sample (after 1 hour of sexual abstinence).

**Variable**	**Total sperm cells motility of the 1st semen sample**	**Total sperm cells motility of the 2nd semen sample**
Sample size (n)	95	95
**Median (%)**	50.00	54.00
95% CI for the median (%)	46.47–56.00	48.47–58.00
Lowest value (%)	0.00	0.00
Highest value (%)	81.00	82.00
IQR (%)	37.25–62.00	43.25–66.00
Hodges-Lehmann median difference (%)	2.00	
95%CI Hodges-Lehmann median difference (%)	–1.50–6.00	
Two-tailed probability	0.2978	

Notes: The groups were compared using the Wilcoxon test (median test).
Abbreviations: 95% CI, 95% confidence interval; IQR, interquartile range; n, number of subjects; p, level of statistical significance of differences between the study groups.

Supplementary Table S9. Descriptive statistics and comparisons of the immotile sperm cells between the 1st semen sample (after 2–7 days of sexual abstinence) and the 2nd semen sample (after 1 hour of sexual abstinence).

Variable	Immotile sperm cells of the 1st semen sample	Immotile sperm cells of the 2nd semen sample
Sample size (n)	95	95
**Median (%)**	50.00	46.00
95% CI for the median (%)	44.00–53.53	42.00–51.53
Lowest value (%)	19.00	18.00
Highest value (%)	100.00	100.00
IQR (%)	38.00–62.75	34.00–56.75
Hodges-Lehmann median difference (%)	−2.00	
95%CI Hodges-Lehmann median difference (%)	−6.00–1.50	
Two-tailed probability	0.2978	

Notes: The groups were compared using the Wilcoxon test (median test).
Abbreviations: 95% CI, 95% confidence interval; IQR, interquartile range; n, number of subjects; p, level of statistical significance of differences between the study groups.

Supplementary Table S10. Descriptive statistics and comparisons of the live (eosin-negative) sperm cells between the 1st semen sample (after 2–7 days of sexual abstinence) and the 2nd semen sample (after 1 hour of sexual abstinence).

Variable	Eosin-negative sperm cells of the 1st semen sample	Eosin-negative sperm cells of the 2nd semen sample
Sample size (n)	95	95
**Median (%)**	74.00	74.00
95% CI for the median (%)	72.00–76.00	72.00–76.53
Lowest value (%)	32.00	39.00
Highest value (%)	90.00	92.00
IQR (%)	65.00–79.00	68.00–81.00
Hodges-Lehmann median difference (%)	0.00	
95%CI Hodges-Lehmann median difference (%)	−2.00–2.50	
Two-tailed probability	0.8593	

Notes: The groups were compared using the Wilcoxon test (median test).
Abbreviations: 95% CI, 95% confidence interval; IQR, interquartile range; n, number of subjects; p, level of statistical significance of differences between the study groups.

Supplementary Table S11. Descriptive statistics and comparisons of the live (HOS test-positive) sperm cells between the 1st semen sample (after 2–7 days of sexual abstinence) and the 2nd semen sample (after 1 hour of sexual abstinence).

Variable	HOS test-positive sperm cells of the 1st semen sample	HOS test-positive sperm cells of the 2nd semen sample
Sample size (n)	73	73
**Median (%)**	71.00	70.00
95% CI for the median (%)	68.65–75.00	68.00–73.00
Lowest value (%)	36.00	43.00
Highest value (%)	90.00	88.00
IQR (%)	66.00–77.00	60.75–77.25
Hodges-Lehmann median difference (%)	–1.00	
95%CI Hodges-Lehmann median difference (%)	–3.00–0.50	
Two-tailed probability	0.1576	

Notes: The groups were compared using the Wilcoxon test (median test).
Abbreviations: 95% CI, 95% confidence interval; IQR, interquartile range; n, number of subjects; p, level of statistical significance of differences between the study groups.

Supplementary Table S12. Descriptive statistics and comparisons of the leukocyte (peroxidase-positive cells) concentration between the 1st semen sample (after 2–7 days of sexual abstinence) and the 2nd semen sample (after 1 hour of sexual abstinence).

Variable	Leukocytes concentration of the 1st semen sample	Leukocytes concentration of the 2nd semen sample
Sample size (n)	95	95
Median (×10^6/mL)	0.25	0.25
95% CI for the median (×10^6/mL)	0.25–0.25	0.25–0.25
Lowest value (×10^6/mL)	0.00	0.00
Highest value (×10^6/mL)	2.75	2.75
IQR (×10^6/mL)	0.1625–0.5000	0.0000–0.5000
Hodges-Lehmann median difference (×10^6/mL)	0.00	
95%CI Hodges-Lehmann median difference (×10^6/mL)	–0.10–0.00	
Two-tailed probability	0.5243	

Notes: The groups were compared using the Wilcoxon test (median test).
Abbreviations: 95% CI, 95% confidence interval; IQR, interquartile range; n, number of subjects; p, level of statistical significance of differences between the study groups.

Supplementary Table S13. Descriptive statistics and comparisons of sperm DNA fragmentation (SDF index) between the 1st semen sample (after 2–7 days of sexual abstinence) and the 2nd semen sample (after 1 hour of sexual abstinence).

Variable	SDF of the 1st semen sample	SDF of the 2nd semen sample
Sample size (n)	94	94
Median (%)	**19.00**	**16.50**
95% CI for the median (%)	17.00–21.00	15.00–17.00
Lowest value (%)	5.00	5.00
Highest value (%)	45.00	51.00
IQR (%)	14.00–24.00	12.00–20.00
Hodges-Lehmann median difference (%)	–2.00	
95%CI Hodges-Lehmann median difference (%)	–3.5—1.00	
Two-tailed probability	**0.0002**	

Notes: The groups were compared using the Wilcoxon test (median test).
Abbreviations: 95% CI, 95% confidence interval; IQR, interquartile range; n, number of subjects; p, level of statistical significance of differences between the study groups.

Supplementary Table S14. Descriptive statistics and comparisons of the aniline blue (AB)-positive sperm cells between the 1st semen sample (after 2–7 days of sexual abstinence) and the 2nd semen sample (after 1 hour of sexual abstinence).

Variable	AB-positive sperm cells of the 1st semen sample	AB-positive sperm cells of the 2nd semen sample
Sample size (n)	85	85
Median (%)	14.00	16.00
95% CI for the median (%)	12.99–18.00	13.99–18.00
Lowest value (%)	2.00	0.00
Highest value (%)	49.00	54.00
IQR (%)	9.00–26.00	9.00–24.50
Hodges-Lehmann median difference (%)	0.50	
95%CI Hodges-Lehmann median difference (%)	–1.00–2.50	
Two-tailed probability	0.4146	

Notes: The groups were compared using the Wilcoxon test (median test).
Abbreviations: 95% CI, 95% confidence interval; IQR, interquartile range; n, number of subjects; p, level of statistical significance of differences between the study groups.

Supplementary Table S15. Descriptive statistics and comparisons of the toluidine blue (TB)-positive sperm between the 1st semen sample (after 2–7 days of sexual abstinence) and the 2nd semen sample (after 1 hour of sexual abstinence).

Variable	TB-positive sperm cells of the 1st semen sample	TB-positive sperm cells of the 2nd semen sample
Sample size (n)	85	85
Median (%)	11.00	10.00
95% CI for the median (%)	8.00–13.00	6.99–14.01
Lowest value (%)	0.00	0.00
Highest value (%)	53.00	53.00
IQR (%)	6.00–19.00	4.00–20.00
Hodges-Lehmann median difference (%)	–0.50	
95%CI Hodges-Lehmann median difference (%)	–2.00–1.00	
Two-tailed probability	0.5151	

Notes: The groups were compared using the Wilcoxon test (median test).

Abbreviations: 95% CI, 95% confidence interval; IQR, interquartile range; n, number of subjects; p, level of statistical significance of differences between the study groups.
